# Assessment of quality of life of the children and parents affected by inborn errors of metabolism with restricted diet: preliminary results of a cross-sectional study

**DOI:** 10.1186/1477-7525-11-158

**Published:** 2013-09-19

**Authors:** Alexandre Fabre, Karine Baumstarck, Aline Cano, Anderson Loundou, Julie Berbis, Brigitte Chabrol, Pascal Auquier

**Affiliations:** 1 Multidisciplinary pediatric unit, Hôpital d’Enfants de la Timone, AP-HM, 27 bd Jean Moulin, Marseille 13385, France; 2 EA3279, Self-perceived Health Assessment Research Unit, School of Medicine, Université de la Méditerranée, 27 bd Jean Moulin, Marseille 13385, France; 3 Centre de Référence des Maladies Héréditaires du Métabolisme, Hôpital d’Enfants de la Timone, AP-HM, 27 bd Jean Moulin, Marseille 13385, France

**Keywords:** Inborn errors of metabolism with restricting dietary, Organic aciduria, Urea cycle defect, Maple syrups urine disease, Quality of life, VSP-A, WHOQOL-BREF

## Abstract

**Introduction:**

The development in therapeutic strategies has increased survival of children affected by inborn errors of metabolism with restricted diet (IEMRD). These diseases have mild- and long-term consequences on the health. Little is known about the impact on the quality of life (QoL) of children and their families. The aims of this study were: to compare the QoL of the children and parents affected by IEMRD with the QoL of the general population and one pathology associated with long-term consequences.

**Patients and methods:**

This cross-sectional study was performed at the French Reference Center for inborn metabolic disorders (Marseille, France). Inclusion criteria were: a child with a diagnosis of organic aciduria, urea cycle defect, or maple syrups urine disease (MSUD). Socio-demographics, clinical data, and QoL were recorded.

**Results:**

Twenty-one of 32 eligible families were included during a planned routine visit. Ten (47%, 95% CI 27-69%) children were affected by organic aciduria, six (29%, 95% CI 10-48%) by urea cycle defects, and five (24%, 95% CI 6-42%) by MSUD. Among the younger children, the general well-being was significantly lower in the children with IEMRD than in the leukemia children (58 ± 16 versus 76 ± 15, p = 0.012), and among the older children, the leisure activities were significantly lower in the children with IEMRD than in the leukemia children (29 ± 18 versus 62 ± 22, p < 10^-3^), while the relationships with teachers were better (76 ± 23 versus 60 ± 23, p = 0.01). The physical QoL score was lower in the parents than in the French norms (66 ± 21 versus 75 ± 1, p = 0.05). Factors modulating QoL were: eating and neurologic disorders, enteral nutrition, and feeding modalities.

**Conclusion:**

The children and the parents of children affected presented altered ‘physical’ and ‘social’ QoL scores compared with the norms and patients with leukemia and their families. Future studies based on larger cohort studies should determine the different weights of potential predictive factors of QoL.

## Introduction

Inborn errors of metabolism (IEM) are a heterogeneous group of genetic diseases that affect metabolic pathways [1]. In this group, certain diseases require diets that include a restrictive lifelong regime when the patient has a stable clinical status and a specific emergency regime when the patient has an unstable clinical status. IEM caused by a deficiency in the activity of an enzyme that is responsible for the catabolism of amino acids or for the urea cycle constitute a group that requires a protein-restricted diet [2], known as IEM with restricted diet (IEMRD). The rapid development of diagnostic and therapeutic possibilities has increased survival and defined IEMRD as chronic illnesses [3].

However, the disease and its treatment have both physical and psychological effects on children and their families: poor growth due to the prolonged restricted diet [4-6], motor disorders [6-8], hepatic disorders [4,9], renal disorders [4,6,10], and neurocognitive disorders [6,8,11,12]. Mild- and long-term consequences of the diseases lead to an interest in assessing quality of life (QoL) and in clinical follow-up for surviving children [13-23].

Among the studies exploring the QoL of IEMRD patients, certain limitations should be mentioned, related to the evaluation of heterogeneous samples in terms of the nature of diseases, the assessment methods, and the treatment modalities. No studies connected children’s and parents’ QoL, and no data related to QoL determinants were available. In this work, we explored the QoL of children affected by three IEMRD conditions that needed the same treatment strategy, combining a long-term hypoproteic diet and an emergency diet in case of metabolic decompensation (due to stress, anesthesia, or infection): organic aciduria, a urea cycle defect, and MSUD. The objectives of our study were i) to compare the QoL levels of the children and parents affected by these three IEMRD conditions with the levels observed in the general population and in another pathology associated with long-term consequences (childhood leukemia survivors), ii) to assess the potential factors modulating the QoL of children and parents, and iii) to assess the relationship between the children’s and parents’ QoL scores.

## Materials and methods

### Study design and population

This study incorporated a cross-sectional design and was performed at the Reference Center for inborn metabolic disorders of a French public teaching hospital (La Timone, Marseille, France).

The inclusion criteria were as follows: a child with a diagnosis of one of three IEMRD conditions (organic aciduria, urea cycle defect, or MSUD), born between 1993 and 2010, with parents or legal guardians authorizing participation in the study. A medical database allowed the identification of eligible children according to the selection criteria. The study was proposed to consecutive parents and/or patients during a planned routine visit.

### Ethic aspects

According to the French law (Article L1121-1, Law n°2011-2012 29 december 2011 - art. 5), ethical approval is not needed for researches in which all actions are performed and products used routinely. This study was conducted in accordance with the Declaration of Helsinki and French Good Clinical Practices. Consent was collected for each eligible family.

### Medical records

From the medical records, the following data were collected: 1. sociodemographic: age and gender of the child; 2. clinical: nature of IEMRD (organic aciduria, urea cycle defect, or MSUD), feeding modalities at inclusion (exclusively oral, home enteral nutrition), eating disorders at inclusion (presence or absence), current gastrostomy, and previous enteral nutrition; and 3. current clinical complications: neurologic (motor, sensory, or cognitive impairment), renal (based on creatine clearance level), cardiac (based on echocardiography), hepatic (biological markers), and protein-related (based on actual protein intake rate).

### Evaluation of QoL

The QoL of the children and adolescents was assessed using the Vécu et Santé Perçue de l’Adolescent et de l’Enfant (VSP-A) questionnaire [24-27]. Two child versions were available: the VSP-Ae, designed to be answered by children aged from 8 to 10 years, and the VSP-A, designed to be answered by adolescents aged from 11 to 17 years. One parent version is available: the VSP-Ap, designed to be answered by the parents of children or adolescents of all ages. The 38-item child version (VSP-Ae), described eight dimensions and an index: relations with parents/family (RFa), body image (BI), vitality (VIT), relations with friends (RFr), general well-being (GWB), leisure activities (LEI), school performance (SCH), and relations with medical staff (RMS). The 39-item adolescent version (VSP-A), described 10 dimensions and an index: relations with parents/family (RFa), body image (BI), vitality (VIT), relations with friends (RFr), psychological well-being (PsWB), physical well-being (PhWB), leisure activities (LEI), school performance (SCH), relations with teachers (RT), and relations with medical staff (RMS). The 37-item parent version (VSP-Ap), described 10 dimensions and an index: relations with parents (Rpa), body image (BI), vitality (VIT), relations with friends (RFr), leisure activities (LEI), psychological well-being (PsWB), physical well-being (PhWB), school performance (SCH); relations with teachers (RT), and relations with medical staff (RMS). All scores range between 0 and 100, with higher scores indicating a better QoL. French norms are available [28]. The scores of childhood leukemia survivors are also available (from the LEA cohort) [29]. These data are the property of one of the author (PA) and their use needs no special permission.

The QoL of the parents was assessed using the French version of the World Health Organization Quality of Life (WHOQOL-BREF) questionnaire, which is a generic questionnaire used worldwide [30,31] that describes four domains: physical health, psychological health, social relationships, and environment. French norms are available only for three domains [32].

### Statistical analysis

Continuous variables were expressed as the means and standard deviations and the medians and ranges. Qualitative variables were expressed as percentages. Nonparametric statistics were employed. The VSP-A scores were compared with the scores obtained from a sample of childhood leukemia survivors [29] and from French controls from a normative sample of 1060 subjects [28]. The WHOQOL-BREF scores of the parents were compared with the scores obtained from French controls from a normative sample of 16000 subjects [32]. Comparisons of mean QoL scores between different subgroups (gender of the child, nature of the IEMRD, feeding modality, eating disorders, other clinical disorders, previous or current enteral nutrition, and current gastrostomy) were performed using Mann–Whitney tests. Correlations between children’s QoL scores and parents’ QoL scores were tested using paired Student’s t-tests. The statistical analyses were performed using the SPSS software package, version 17.0 (SPSS Inc., Chicago, IL, USA). All tests were two-sided. Statistical significance was defined as p < 0.05.

## Results

### Sample

Among 32 eligible families, 11 were non-respondents due to abortive contact and refusal to participate. The respondents and non-respondents did not differ according to age or the nature of the IEMRD. Ten children were affected by organic aciduria, six by urea cycle defects, and five by MSUD. The mean age was 98.7 months (SD: 67.1). At the time of evaluation, 12 children were fed exclusively orally, nine received home enteral nutrition, and eight had a gastrostomy. Disorders of any nature (neurologic, renal, cardiac, and hepatic) were reported in 62% of the children. All of the characteristics are detailed in Table 1. Ten children specified their QoL (six using VSP-A and four using VSP-Ae). Fourteen parents specified the QoL of their children using the VSP-Ap, and 21 parents specified their own QoL using the WHOQOL.

**Table 1:** Patients characteristics

**1. Children**	**N = 21**	**N (%)**
Sex	Boys	10 (47.6)
	Girls	11 (52.4)
Age (years)	Mean ± SD	8.25 ± 5.60
	M (min-max)	8.6 (1–18)
Nature of IEMRD	Organic aciduria	10 (47.6)
	Urea cycle defect	6 (28.6)
	MSUD	5 (23.8)
Feeding modality at inclusion	Exclusively oral	12 (57.1)
	Oral and gastrostomy	9 (42.9)
Eating disorders at inclusion		6 (28.5)
Neurologic disorder at inclusion		10 (47.6)
Renal/cardiac/hepatic disorders at inclusion		9 (42.9)
Enteral nutrition		15 (71.4)
Current gastrostomy		8 (38.1)
Protein (Gr/kg/j)	Mean ± SD	1.07 ± 0.32
	M (min-max)	1.14 (0.18-1.49)
Natural protein (Gr/kg/j)	Mean ± SD	0.67 ± 0.29
	M (min-max)	0.71 (0.17-1.16)
**2. Parents**	**N = 21**	
Age (years)	Mean ± SD	39.14 ± 6.64
	M (min-max)	39 (30–58)
Occupational status	Yes	11 (52.4)
	No	10 (47.6)

*IEMRD:* Inborn errors of metabolism with restricting dietary.

*MSUD:* maple syrup urine disease.

*SD* standard deviation, M median, min-max minimum and maximum.

### QoL of IEMRD sample compared with general population and leukemia

#### QoL of children and adolescents

##### - QoL of children from self-report

**Figure 1:**
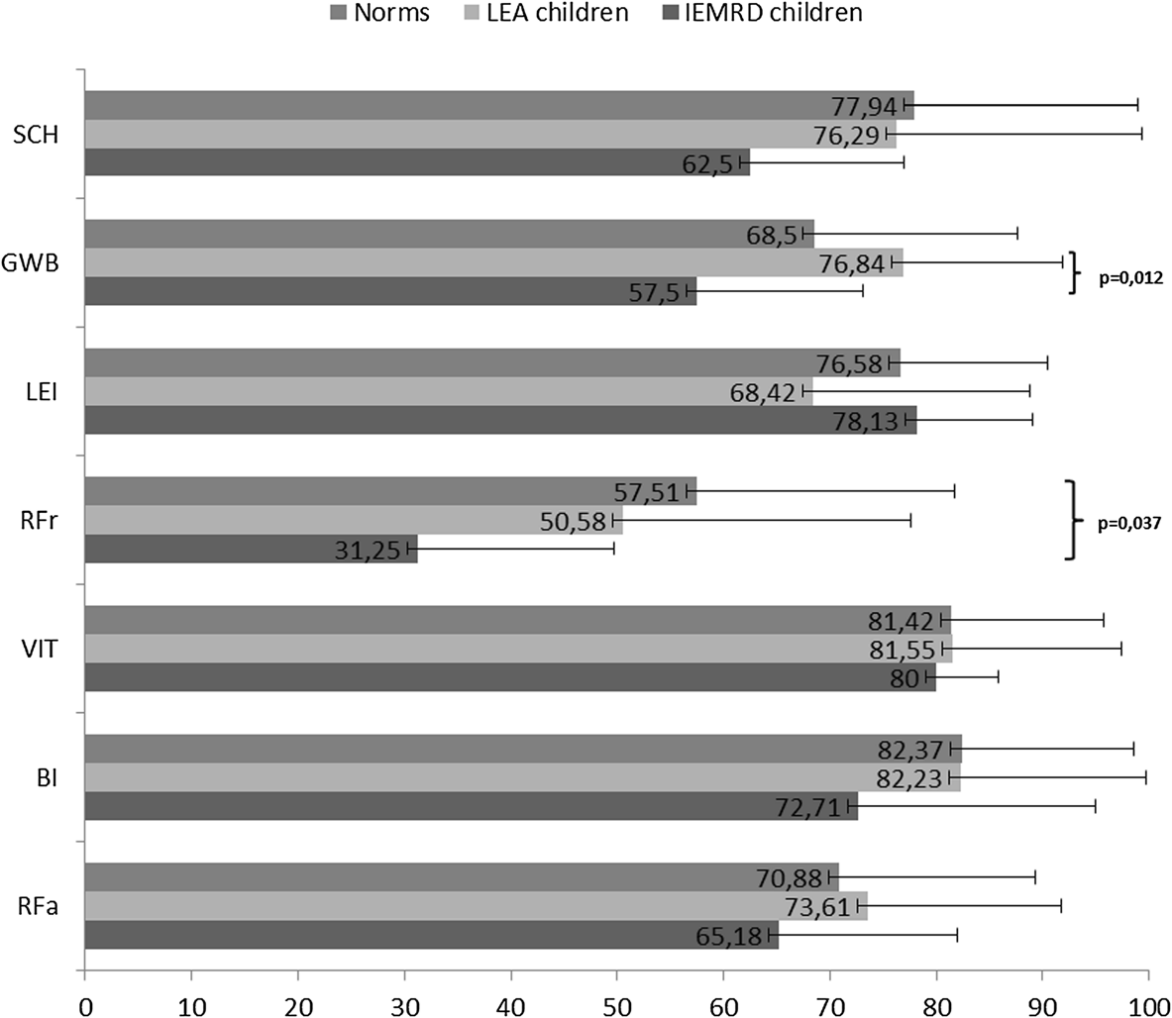
QoL children (8–10 years): comparisons of dimensions scores between the IEMRD children and LEA cohort and French norms.

The QoL scores of the children aged from 8 to 10 years are shown in Figure 1. The relations with friends was the dimension with the most altered score, and the vitality dimension score was the least altered. The scores of the children with IEMRD were always lower than the scores of the long-term survivors of childhood leukemia (LEA cohort) and the norms, except for the leisure activities dimension. These differences were not significant, except for the general well-being score, which was significantly lower in the children with IEMRD than in the LEA children (p = 0.012), and for the relations with friends score, which was lower in the children with IEMRD than in the norms (p = 0.037).

**Figure 2:**
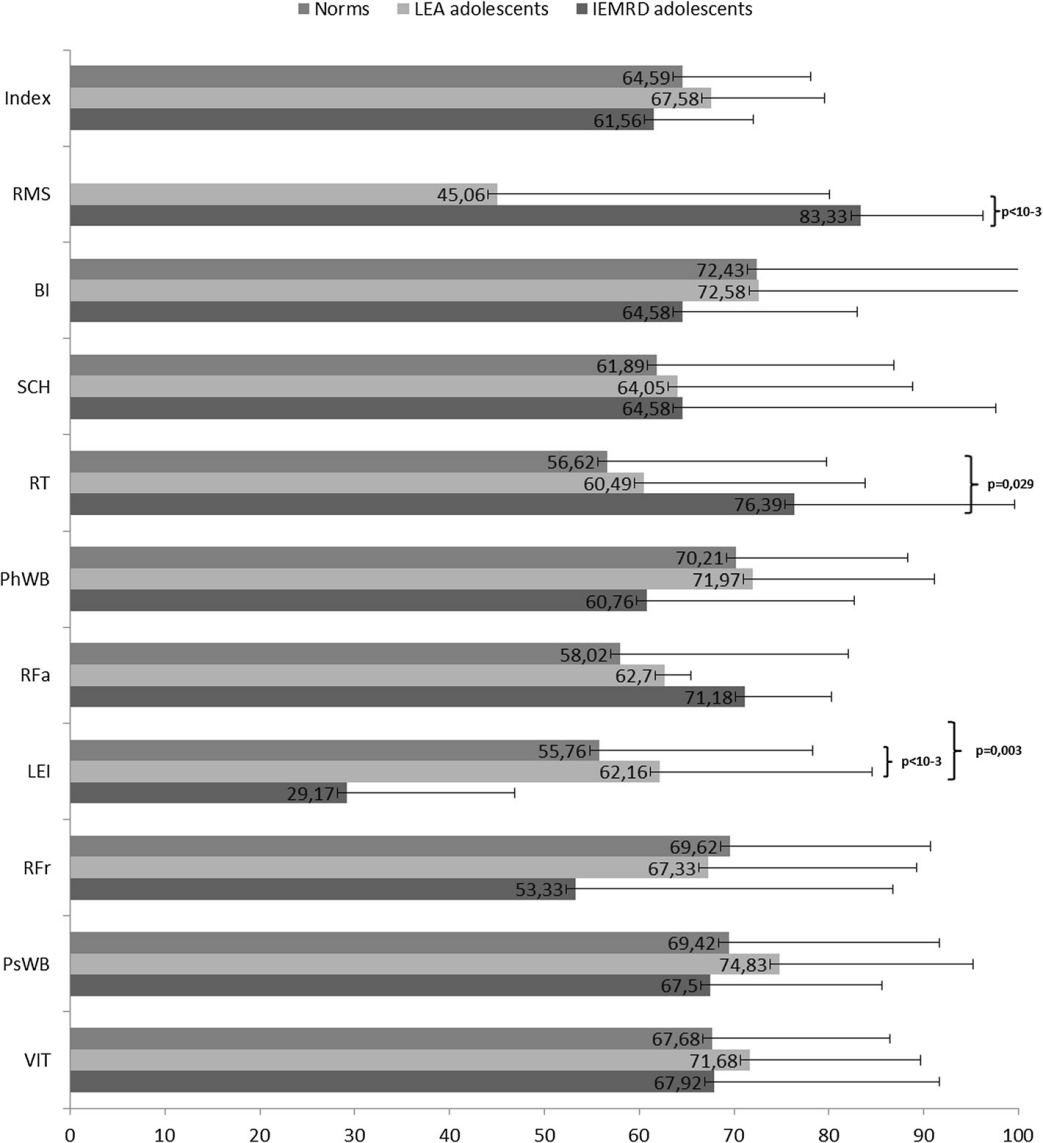
QoL adolescents (11–18 years): comparisons of dimensions scores between the IEMRD adolescents and LEA cohort and French norms.

The QoL scores of the adolescents over 11 years of age are shown in Figure 2. Leisure was the dimension with the most altered score, and the ‘social’ dimension scores were the least altered. The scores of the adolescents with IEMRD were significantly lower for the leisure activities dimension than the scores of the LEA cohort adolescents and norms (p = 0.001 and p = 0.003, respectively). In contrast, the adolescents with IEMRD scored better than the norms for the relations with teachers (p = 0.029) and better than the LEA adolescents for the relations with medical staff (p < 0.001).

##### - QoL of children from parents’ reports

**Figure 3:**
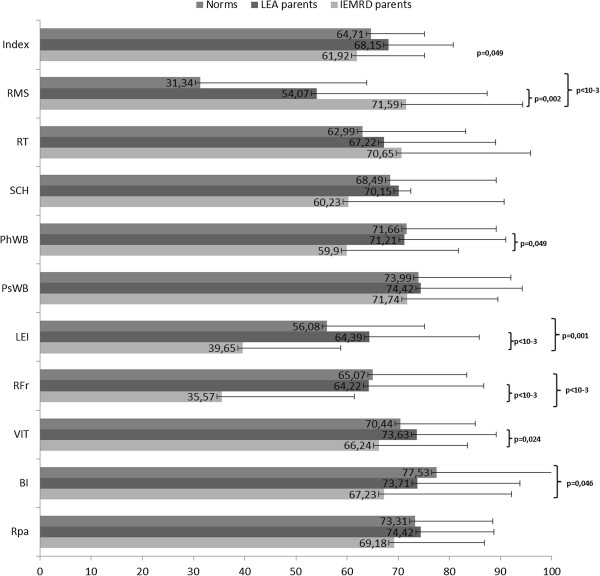
Children’ QoL reported by parents (VSP-Ap): comparisons of scores between the IEMRD sample and LEA cohort and norms.

The QoL scores of children based on their parents’ views are reported in Figure 3. Leisure and relations with friends were the most affected dimensions, whereas the scores for relations with teachers and medical staff were high. Compared with the survivors of childhood leukemia, the children with IEMRD assessed by their parents scored significantly lower for the index (p = 0.049) and four ‘social’ and ‘physical’-like dimensions: physical well-being, leisure activities, relations with friends, and vitality (p = 0.007, p < 0.001, p < 0.001, and p = 0.024, respectively). However, the parents scored higher for the relations with medical staff (p = 0.002). Compared with the French norms, the scores for leisure activities, relations with friends, and body image were significantly lower for the children with IEMRD (p = 0.001, p < 0.001, and p = 0.046, respectively).

#### QoL of parents

**Figure 4:**
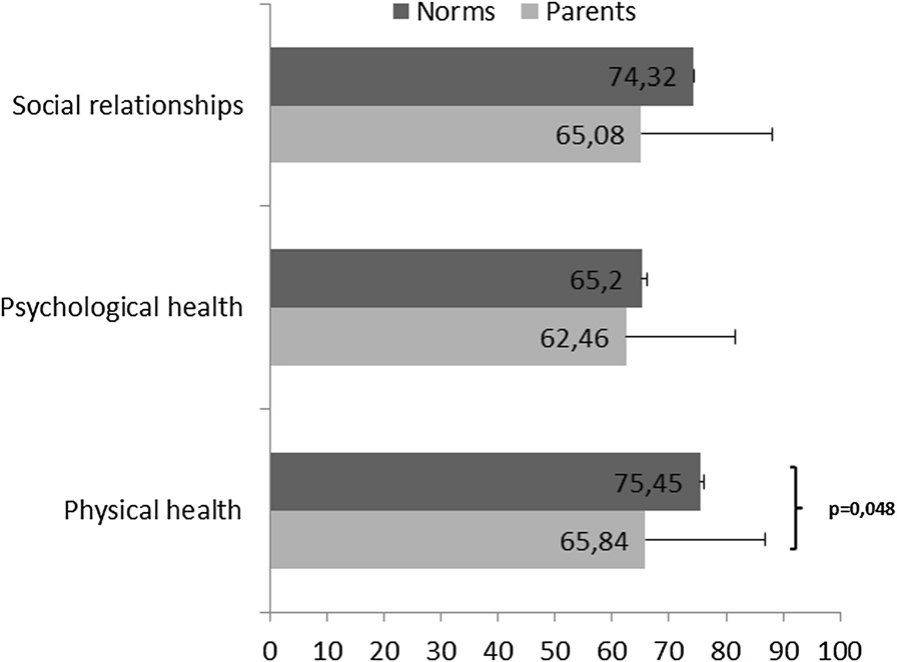
**Parents’ QoL: comparisons of WHOQOL scores between the IEMRD parents and norms.**

The QoL dimension scores were lower in the parents of children with IEMRD than in the French norms. The difference was only significant for the physical health score (p < 0.05) (Figure 4).

### Factors modulating QoL of children with IEMRD and their parents

The results for the factors modulating the QoL of the children with IEMRD are provided in Additional file 1. The children affected by urea cycle defects had lower scores than children affected by the other two IEMRD conditions, especially for the relationships with family and body image dimensions. The vitality score was lower for the children presenting with eating and neurologic disorders. The score for psychological well-being was higher for the children who reported receiving enteral nutrition in the past. The index was not impacted by the various sociodemographic and clinical parameters.

The results for the factors modulating the QoL of the parents of children with IEMRD are shown in Additional file 2. The WHOQOL scores did not differ according to the sex of the child, nature of the IEMRD, feeding modality at inclusion, or eating disorders. The parents of children affected by neurologic disorders reported significantly altered social relationships. The parents of children with a current gastrostomy reported higher scores for two dimensions: physical health and social relationships.

### Relationships between QoL assessments of parents and children

The results of the VSP-Ap (children’s QoL assessed by the parents) and VSP-A/VSP-Ae (children’s QoL assessed by the children themselves) only differed for the vitality score, which was lower in the parents’ assessment (Additional file 3).

## Discussion

An understanding of the impact of pediatric chronic illnesses on QoL is important for the health care workers who manage the relevant populations. We present one of the first studies to specifically assess children with IEMRD who are at risk of acute decompensation and their parents. Consistent with previous studies, our findings confirm that IEMRD impact the QoL of both parents and children affected by IEMRD [20,33,34]. One publication explored the QoL of children with maple syrup urine disease (MSUD), showing altered QoL scores compared with controls and similar QoL scores as children with cancer [33]. Among the studies exploring the QoL of the parents, lower scores were found than for the parents of children affected by other chronic illnesses, such as sickle cell disease or Down syndrome, or for controls [20,23,34].

However, our results show that the ‘physical-like’ aspects of QoL are more impacted than the ‘psychological-like’ aspects, both for parents and for children. Indeed, the parents of these children reported lower scores on the physical dimension of the WHOQOL than the controls, and the children reported lower ‘physical’ scores than leukemia survivors. One explanation is the well-known phenomena of coping strategies and adjustment developed by patients and/or families [35]. Relations with friends and leisure activities were also defined by the children as affected domains. We hypothesize that the dietary regime constraints are partly responsible for these affects, as shown in the comment that food preparation with minute weighting and logistics are problematic. Even if a parent stated always being prepared for potential decompensation, he or she also asserted that it can be hard to prepare for leisure and holidays. The limited diet was a complaint shared by the parents and the children. Alimentation plays a large cultural role; for example, parents complained that certain aliment cannot be shared, as these limitations are without exception. Moreover, in certain cases, it was noted that there are few pleasures in consuming food. These limits remain constant; indeed, in one study of approximately eight adults affected with MSUD and their parents, the dietary constraint was one of the major themes [18].

In contrast, the children reported better relationships with medical staff than did the leukemia survivors. In comparison with other chronic illnesses, such as cancer, IEMRD has specific features. The disease is lifelong, as opposed to cancer, for which the conventional treatment does not cure the disease. There is the lifelong strain of dietary management. Families and children face ongoing metabolic decompensation and a risk of death with decompensation and regularly visit hospitals and physicians throughout childhood. For all of the above reasons, we hypothesize that the parents and their children have faith in and rely on medical staff. Other authors did not report this phenomenon [33], which can be explained by the specifics of the health care system. The French free health care system most likely allows parents to not be worried by financial problems.

Interestingly, the presence of gastrostomy can have certain ‘protective effects’ on the self-reported QoL of parents, notably in the physical health and social relationship domains. A similar effect was previously shown [36], for children with inherited metabolic diseases [34] and cerebral palsy [37,38]. Many explanations should be discussed. First, in case of enteral nutrition, gastrostomy removes the necessity of an enteral feeding tube, which is more apparent and less stable. Moreover, gastrostomy can be easily used in cases of difficulties in eating. Second, it has been previously reported that gastrostomy can decrease parents’ stress [34,39]. Gastrostomy did not have an influence on children’s QoL, as found for children with cerebral palsy [40]. In our cohort, the patients with urea cycle defects seemed to have a lower QoL, as reported by their parents. The influence of the nature of IEM was not explored in the previous studies [34]. This finding could be related to the more severe natural course of the urea cycle defects, but we cannot exclude the effect of the size of the study population.

Two limitations of our study must be mentioned:

i. Moderate associations were possibly missed because of low statistical power due to the sample size, which was arguably too small. The sample size did not allow a multivariate approach that takes into account the potential confounding factors. A larger sample will allow the confirmation of these findings.

ii. Cross-sectional studies examine individuals with heterogeneous disease durations. Longitudinal studies provide more valid information and are necessary to more accurately determine the weights of potential predictive factors of QoL. Future studies based on cohort studies should provide more robust findings, particularly regarding the childhood/adulthood transition, and should propose appropriate treatment strategies.

## Conclusion

The children and the parents of children affected with organic aciduria, urea cycle defects, or MSUD presented altered ‘physical’ and ‘social’ QoL scores compared with the norms and patients with leukemia and their families. Future studies based on larger cohort studies should provide supplementary findings to validate these preliminary results and determine the different weights of potential predictive factors of QoL, allowing the adaptation of appropriate care strategies for children and their caregivers. Gastrostomy, when required, can provide a degree of help to the parents.

## Competing interests

The authors declare that they have no competing interest.

## Authors’ contributions

Conception and design: AF, BC, PA. Study coordination: AF, AC, BC, PA. Inclusion and clinical data collection: AF, AC, BC. Analysis of data: KB, AL. Interpretation of data: AF, KB, JB, PA. Drafting and writing of manuscript: AF, KB, PA. Revision of manuscript: AF, KB, AC, AL, JB, BC, PA. Given final approval of the manuscript: AF, KB, AC, AL, JB, BC, PA.

## Supplementary Material

Additional file 1 Associations between children QoL reported by parents and characteristics of children (n = 14). Click here for file

Additional file 2 Associations between parents QoL and characteristics of children (n = 21). Click here for file

Additional file 3 Children QoL: relations between scores reported by children and parents (n = 10). Click here for file

## References

[B1] Saudubray JM Sedel F Chabrol B, De Lonlay P Classification et circonstance de découverte des maladies héréditaires du métabolisme Progrés en pédiatrie- Maladies métaboliques Héréditaires 2011 1 10

[B2] de Baulny HO Benoist JF Rigal O Touati G Rabier D Saudubray JM Methylmalonic and propionic acidaemias: management and outcome J Inherit Metab Dis 2005 28 3 415 423 10.1007/s10545-005-7056-1 15868474

[B3] Cederbaum S Lemons C Batshaw ML Alternative pathway or diversion therapy for urea cycle disorders now and in the future Mol Genet Metab 2010 100 3 219 220 10.1016/j.ymgme.2010.04.008 20462778

[B4] Rake JP Visser G Labrune P Leonard JV Ullrich K Smit GP Guidelines for management of glycogen storage disease type I - European Study on glycogen storage disease type I (ESGSD I) Eur J Pediatr 2002 161 Suppl 1 S112 S119 12373584 10.1007/s00431-002-1016-7

[B5] Acosta PB Yannicelli S Ryan AS Arnold G Marriage BJ Plewinska M Bernstein L Fox J Lewis V Miller M Nutritional therapy improves growth and protein status of children with a urea cycle enzyme defect Mol Genet Metab 2005 86 4 448 455 10.1016/j.ymgme.2005.08.012 16260164

[B6] Horster F Baumgartner MR Viardot C Suormala T Burgard P Fowler B Hoffmann GF Garbade SF Kolker S Baumgartner ER Long-term outcome in methylmalonic acidurias is influenced by the underlying defect (mut0, mut-, cblA, cblB) Pediatr Res 2007 62 2 225 230 10.1203/PDR.0b013e3180a0325f 17597648

[B7] Waggoner DD Buist NR Donnell GN Long-term prognosis in galactosaemia: results of a survey of 350 cases J Inherit Metab Dis 1990 13 6 802 818 10.1007/BF01800204 1706789

[B8] le Roux C Murphy E Hallam P Lilburn M Orlowska D Lee P Neuropsychometric outcome predictors for adults with maple syrup urine disease J Inherit Metab Dis 2006 29 1 201 202 10.1007/s10545-006-0223-1 16601892

[B9] Masurel-Paulet A Poggi-Bach J Rolland MO Bernard O Guffon N Dobbelaere D Sarles J de Baulny HO Touati G NTBC treatment in tyrosinaemia type I: long-term outcome in French patients J Inherit Metab Dis 2008 31 1 81 87 10.1007/s10545-008-0793-1 18214711

[B10] Cosson MA Benoist JF Touati G Dechaux M Royer N Grandin L Jais JP Boddaert N Barbier V Desguerre I Long-term outcome in methylmalonic aciduria: a series of 30 French patients Mol Genet Metab 2009 97 3 172 178 10.1016/j.ymgme.2009.03.006 19375370

[B11] Bachmann C Long-term outcome of patients with urea cycle disorders and the question of neonatal screening Eur J Pediatr 2003 162 Suppl 1 S29 S33 14634803 10.1007/s00431-003-1347-z

[B12] Kyllerman M Skjeldal O Christensen E Hagberg G Holme E Lonnquist T Skov L Rotwelt T von Dobeln U Long-term follow-up, neurological outcome and survival rate in 28 Nordic patients with glutaric aciduria type 1 Eur J Paediatr Neurol 2004 8 3 121 129 10.1016/j.ejpn.2003.12.007 15120683

[B13] Bosch AM Grootenhuis MA Bakker HD Heijmans HS Wijburg FA Last BF Living with classical galactosemia: health-related quality of life consequences Pediatrics 2004 113 5 e423 e428 10.1542/peds.113.5.e423 15121984

[B14] Bosch AM Tybout W van Spronsen FJ de Valk HW Wijburg FA Grootenhuis MA The course of life and quality of life of early and continuously treated Dutch patients with phenylketonuria J Inherit Metab Dis 2007 30 1 29 34 10.1007/s10545-006-0433-6 17160615

[B15] Bosch M van der Weijden T Grol R Schers H Akkermans R Niessen L Wensing M Structured chronic primary care and health-related quality of life in chronic heart failure BMC Health Serv Res 2009 9 104 10.1186/1472-6963-9-104 19545385 PMC2710325

[B16] Cederbaum JA LeMons C Rosen M Ahrens M Vonachen S Cederbaum SD Psychosocial issues and coping strategies in families affected by urea cycle disorders J Pediatr 2001 138 1 Suppl S72 S80 11148552 10.1067/mpd.2001.111839

[B17] Lambert C Boneh A The impact of galactosaemia on quality of life–a pilot study J Inherit Metab Dis 2004 27 5 601 608 15669675 10.1023/b:boli.0000042957.98782.e4

[B18] Packman W Mehta I Rafie S Mehta J Naldi M Mooney KH Young adults with MSUD and their transition to adulthood: psychosocial issues J Genet Couns 2012 21 5 692 703 10.1007/s10897-012-9490-1 22350623

[B19] Gassio R Campistol J Vilaseca MA Lambruschini N Cambra FJ Fuste E Do adult patients with phenylketonuria improve their quality of life after introduction/resumption of a phenylalanine-restricted diet? Acta Paediatr 2003 92 12 1474 1478 10.1111/j.1651-2227.2003.tb00834.x 14971801

[B20] Hatzmann J Heymans HS Ferrer-i-Carbonell A van Praag BM Grootenhuis MA Hidden consequences of success in pediatrics: parental health-related quality of life–results from the care project Pediatrics 2008 122 5 e1030 e1038 10.1542/peds.2008-0582 18852185

[B21] Landolt MA Nuoffer JM Steinmann B Superti-Furga A Quality of life and psychologic adjustment in children and adolescents with early treated phenylketonuria can be normal J Pediatr 2002 140 5 516 521 10.1067/mpd.2002.123663 12032515

[B22] Simon E Schwarz M Roos J Dragano N Geraedts M Siegrist J Kamp G Wendel U Evaluation of quality of life and description of the sociodemographic state in adolescent and young adult patients with phenylketonuria (PKU) Health Qual Life Outcomes 2008 6 25 10.1186/1477-7525-6-25 18366761 PMC2329607

[B23] Ten Hoedt AE Maurice-Stam H Boelen CC Rubio-Gozalbo ME Van Spronsen FJ Wijburg FA Bosch AM Grootenhuis MA Parenting a child with phenylketonuria or galactosemia: implications for health-related quality of life J Inherit Metab Dis 2011 34 2 391 398 10.1007/s10545-010-9267-3 21290186 PMC3063540

[B24] Simeoni MC Auquier P Antoniotti S Sapin C San Marco JL Validation of a French health-related quality of life instrument for adolescents: the VSP-A Qual Life Res 2000 9 4 393 403 10.1023/A:1008957104322 11131932

[B25] Auquier P Clément A Sapin C El Khammar M San Marco JL Siméoni MC Development, validation of HRQL measurement in children: VSP-Ae Qual Life Res 2001 10 268

[B26] Simeoni MC Sapin C Antoniotti S Auquier P Health-related quality of life reported by French adolescents: a predictive approach of health status? J Adolesc Health 2001 28 4 288 294 10.1016/S1054-139X(00)00198-1 11287246

[B27] Sapin C Simeoni MC El Khammar M Antoniotti S Auquier P Reliability and validity of the VSP-A, a health-related quality of life instrument for ill and healthy adolescents J Adolesc Health 2005 36 4 327 336 10.1016/j.jadohealth.2004.01.016 15780788

[B28] Ravens-Sieberer U Auquier P Erhart M Gosch A Rajmil L Bruil J Power M Duer W Cloetta B Czemy L The KIDSCREEN-27 quality of life measure for children and adolescents: psychometric results from a cross-cultural survey in 13 European countries Qual Life Res 2007 16 8 1347 1356 10.1007/s11136-007-9240-2 17668292

[B29] Michel G Bordigoni P Simeoni MC Curtillet C Hoxha S Robitail S Thuret I Pall-Kondolff S Chambost H Orbicini D Health status and quality of life in long-term survivors of childhood leukaemia: the impact of haematopoietic stem cell transplantation Bone Marrow Transplant 2007 40 9 897 904 10.1038/sj.bmt.1705821 17704791

[B30] Power M Harper A Bullinger M The world health organization WHOQOL-100: tests of the universality of quality of life in 15 different cultural groups worldwide Health Psychol 1999 18 5 495 505 10519466 10.1037//0278-6133.18.5.495

[B31] WHOQOL: Group Development of the world health organization WHOQOL-BREF quality of life assessment. The WHOQOL group Psychol Med 1998 28 3 551 558 9626712 10.1017/s0033291798006667

[B32] Baumann C Erpelding ML Regat S Collin JF Briancon S The WHOQOL-BREF questionnaire: French adult population norms for the physical health, psychological health and social relationship dimensions Rev Epidemiol Sante Publique 2010 58 1 33 39 10.1016/j.respe.2009.10.009 20096515

[B33] Packman W Henderson SL Mehta I Ronen R Danner D Chesterman B Packman S Psychosocial issues in families affected by maple syrup urine disease J Genet Couns 2007 16 6 799 809 10.1007/s10897-007-9114-3 17703353

[B34] Hatzmann J Valstar MJ Bosch AM Wijburg FA Heymans HS Grootenhuis MA Predicting health-related quality of life of parents of children with inherited metabolic diseases Acta Paediatr 2009 98 7 1205 1210 10.1111/j.1651-2227.2009.01269.x 19397532

[B35] Rapkin BD Schwartz CE Toward a theoretical model of quality-of-life appraisal: Implications of findings from studies of response shift Health Qual Life Outcomes 2004 15 1 12 10.1186/1477-7525-2-14 PMC408464 15023229

[B36] Avitsland TL Kristensen C Emblem R Veenstra M Mala T Bjornland K Percutaneous endoscopic gastrostomy in children: a safe technique with major symptom relief and high parental satisfaction J Pediatr Gastroenterol Nutr 2006 43 5 624 628 10.1097/01.mpg.0000229550.54455.63 17130739

[B37] Sullivan PB Juszczak E Bachlet AM Thomas AG Lambert B Vernon-Roberts A Grant HW Eltumi M Alder N Jenkinson C Impact of gastrostomy tube feeding on the quality of life of carers of children with cerebral palsy Dev Med Child Neurol 2004 46 12 796 800 15581151 10.1017/s0012162204001392

[B38] Smith SW Camfield C Camfield P Living with cerebral palsy and tube feeding: a population-based follow-up study J Pediatr Gastroenterol Nutr 1999 135 307 310 10.1016/s0022-3476(99)70125-3 10484794

[B39] Avitsland TL Faugli A Pripp AH Malt UF Bjornland K Emblem R Maternal psychological distress and parenting stress after gastrostomy placement in children J Pediatr Gastroenterol Nutr 2012 55 5 562 566 10.1097/MPG.0b013e31826078bd 22644463

[B40] Mahant S Friedman JN Connolly B Goia C Macarthur C Tube feeding and quality of life in children with severe neurological impairment Arch Dis Child 2009 94 9 668 673 10.1136/adc.2008.149542 19465586

