# Supplementary material for: Assessment of quality of life of the children and parents affected by inborn errors of metabolism with restricted diet: preliminary results of a cross-sectional study
Source: Health Qual Life Outcomes. 2013 Sep 19;11:158. doi: 10.1186/1477-7525-11-158 (PMC3848736; doi:10.1186/1477-7525-11-158)
Supplement: Additional file 1 — Associations between children QoL reported by parents and characteristics of children (n = 14). [file 1477-7525-11-158-S1.docx]

**Additional file 1. Associations between children QoL reported by parents and characteristics of children (n=14)**

| **VSP-Ap** | **RFa** | **BI** | **VI** | **RFr** | **LEI** | **PsWB** | **PhWB** | **Sch** | **RTe** | **RMS** | **Index** |
| --- | --- | --- | --- | --- | --- | --- | --- | --- | --- | --- | --- |
| Sex of the child |  |  |  |  |  |  |  |  |  |  |  |
| Boys | 72,32±12,65 | 71,42±27,15 | 62,05±20,20 | 33,33±28,22 | 38,80±21,76 | 67,26±25,39 | 55,35±29,63 | 50±36,22 | 66,66±22,97 | 70,23±17,90 | 63,08±13,98 |
| Girls | 66,07±15,85 | 61,78±27,47 | 65,32±17,16 | 35,71±23,52 | 39,28±6,725 | 71,07±16,02 | 61,60±25,36 | 60,71±33,40 | 69,04±36,23 | 70,13±27,69 | 58,84±18,48 |
| p-value | NS | NS | NS | NS | NS | NS | NS | NS | NS | NS | NS |
| Nature of the IEMRD |  |  |  |  |  |  |  |  |  |  |  |
| Organic aciduria | 76,30±10,26 | 79,68±18,95 | 68,09±18,33 | 36,25±25,73 | 39,37±20,25 | 73,33±17,68 | 57,03±32,29 | 62,5±28,34 | 82,14±17,63 | 68,75±18,76 | 66,68±10,20 |
| Urea cycle defect | 51,38±15,77 | 28,88±11,34 | 47,91±18,83 | 7,5±3,54 | 41,66±7,637 | 60,55±19,65 | 45,83±18,04 | 43,75±61,87 | 44,44±50,91 | 66,66±38,18 | 42,70±29,78 |
| MSUD | 68,05±2,41 | 69,44±20,55 | 67,70±10,97 | 48,33±17,55 | 35,55±5,853 | 66,66±32,54 | 75±0 | 45,83±40,18 | 58,33±8,333 | 81,25±8,838 | 58,12±10,84 |
| p-value | **0.035** | **0.029** | NS | NS | NS | NS | NS | NS | NS | NS | NS |
| Feedings modality at inclusion | |  |  |  |  |  |  |  |  |  |  |
| Exclusively oral | 67,70±16,32 | 62,39±26,54 | 68,75±20,25 | 41,87±24,04 | 44,58±14,19 | 69,06±24,15 | 62,5±23,62 | 48,43±33,69 | 68,75±34,14 | 75,59±23,86 | 59,95±19,55 |
| Mixt | 71,18±11,81 | 72,22±28,34 | 56,94±13,46 | 23±23,34 | 31,66±15,05 | 69,30±16,58 | 53,12±31,80 | 67,5±33,77 | 66,66±24,29 | 63,88±19,48 | 62,19±8,82 |
| p-value | NS | NS | NS | NS | NS | NS | NS | NS | NS | NS | NS |
| Eating disorders at inclusion | |  |  |  |  |  |  |  |  |  |  |
| No | 71,66±15,81 | 67,83±28,28 | 68,22±18,60 | 39,5±25,97 | 43,16±14,15 | 65,16±22,06 | 60,62±28,57 | 55±33,95 | 71,66±31,72 | NA | 73,14±22,73 |
| Yes | 61,80±7,88 | 58,33±29,16 | 46,87±5,412 | 12,5±3,54 | 28,33±18,92 | 76,38±14,63 | 45,83±23,66 | 50±53,03 | 54,16±29,46 |  | 55,55±17,34 |
| p-value | NS | NS | **0.041** | NS | NS | NS | NS | NS | NS |  | NS |
| Neurologic disorders at inclusion | |  |  |  |  |  |  |  |  |  |  |
| No | 75±9,13 | 69,44±22,77 | 75,17±9,28 | 44,16±20,59 | 46,66±15,05 | 73,47±7,367 | 66,66±21,16 | 66,66±23,27 | 83,33±19,00 | 78,33±18,25 | 67,80±4,705 |
| Yes | 64,84±16,19 | 64,47±30,70 | 55,07±18,67 | 26,42±26,41 | 33,33±14,00 | 65,93±26,71 | 52,34±30,05 | 46,42±39,99 | 54,76±31,86 | 65,10±23,56 | 54,90±19,99 |
| p-value | NS | NS | **0.032** | NS | NS | NS | NS | NS | NS | NS | NS |
| Renal/cardiac/hepatic disorders at inclusion | | |  |  |  |  |  |  |  |  |  |
| No | 65,47±16,79 | 57,61±30,54 | 61,75±19,61 | 40,83±23,54 | 46,66±14,24 | 60,35±19,73 | 58,03±21,56 | 43,75±40,11 | 59,52±36,14 | 68,45±22,80 | 57,37±19,25 |
| Yes | 72,91±10,95 | 75,59±20,61 | 65,62±17,76 | 29,28±26,20 | 31,42±13,45 | 77,97±18,43 | 58,92±32,84 | 66,07±25,73 | 77,77±18,00 | 72,22±22,77 | 64,84±11,56 |
| p-value | NS | NS | NS | NS | NS | 0.047 | NS | NS | NS | NS | NS |
| Previous enteral nutrition | |  |  |  |  |  |  |  |  |  |  |
| No | 50±23,57 | 32,91±18,26 | 42,18±19,88 | 35±42,42 | 40,83±1,178 | 34,58±7,660 | 50±35,35 | NA | 25±35,35 | 50±35,35 | 36,05±20,37 |
| Yes | 71,87±9,78 | 74,16±24,04 | 65,10±15,58 | 35,55±24,16 | 35,5±13,63 | 74,91±16,92 | 59,37±29,35 |  | 73,14±23,11 | 69,90±18,13 | 65,59±9,761 |
| p-value | NS | NS | NS | NS | NS | **0.031** | NS |  | NS | NS | NS |
| Current gastrostomy |  |  |  |  |  |  |  |  |  |  |  |
| No | 63,54±17,16 | 62,36±28,50 | 60,93±23,69 | 42,5±27,70 | 39,44±11,18 | 65±26,74 | 60,41±27,00 | 31,25±32,35 | 63,33±41,49 | 60±25,27 | 54,17±25,36 |
| Yes | 74,16±10,37 | 70,83±31,45 | 60,20±12,11 | 26,25±25,61 | 34±15,57 | 68,16±18,28 | 51,25±35,18 | 81,25±16,13 | 66,66±24,29 | 68,33±18,06 | 62,19±8,82 |
| p-value | NS | NS | NS | NS | NS | NS | NS | NS | NS | NS | NS |

RFa Relationships with parents/family, BI Body image, VI Vitality, RFr Relationships with friends, LEI Leisures, PsWB Psychological well-being, PhWB Physical well-being, Sch School performance, RTe Relationships with teachers, RMS Relationships with medical staff

Higher the scores, higher the QoL level

Bold values: p-value < 0.05

NA non available (dimenion score not calculated)
