# Supplementary material for: Assessment of quality of life of the children and parents affected by inborn errors of metabolism with restricted diet: preliminary results of a cross-sectional study
Source: Health Qual Life Outcomes. 2013 Sep 19;11:158. doi: 10.1186/1477-7525-11-158 (PMC3848736; doi:10.1186/1477-7525-11-158)
Supplement: Additional file 2 — Associations between parents QoL and characteristics of children (n = 21). [file 1477-7525-11-158-S2.docx]

**Additional file 2. Associations between parents QoL and characteristics of children (n=21)**

| **WHOQOL** | **Physical health** | **Psychological health** | **Social relationships** | **Environment** |
| --- | --- | --- | --- | --- |
| Sex of the child |  |  |  |  |
| Boys | 67.08 ± 20.54 | 63.75 ± 19.88 | 70.83 ± 21.87 | 57.93 ± 21.88 |
| Girls | 64.71 ± 22.60 | 61.29 ± 19.42 | 59.84 ± 23.81 | 69.93 ± 16.87 |
| p-value | 1.000 | 0.620 | 0.242 | 0.157 |
| Nature of the IEMRD |  |  |  |  |
| Organic aciduria | 65,65±19,78 | 59,83±18,55 | 67,5±22,29 | 58,97±24,63 |
| Urea cycle defect | 77,57±17,22 | 68,88±12,98 | 69,44±27,21 | 73,95±7,306 |
| MSUD | 52,14±23,22 | 60±27,73 | 55±20,91 | 63,00±18,20 |
| p-value | 0.156 | 0.630 | 0.396 | 0.423 |
| Feedings modality |  |  |  |  |
| Exclusively oral | 58.73 ± 21.89 | 59.03 ± 19.61 | 61.46 ± 26.30 | 60.03 ± 20.11 |
| Mixt | 75.33 ± 16.73 | 67.03 ± 18.71 | 69.91 ± 18.13 | 69.79 ± 19.26 |
| p-value | 0.087 | 0.253 | 0.453 | 0.392 |
| Eating disorders |  |  |  |  |
| No | 66.30 ± 20.73 | 64.68 ± 15.90 | 65.71 ± 23.33 | 66.95 ± 21.36 |
| Yes | 60.61 ± 24.71 | 52.08 ± 24.61 | 57.64 ± 24.21 | 56.25 ± 18.75 |
| p-value | 0.598 | 0.311 | 0.537 | 0.186 |
| Neurologic disorders |  |  |  |  |
| No | 71.54 ± 22.42 | 66.14 ± 16.88 | 76.52 ± 18.47 | 69.56 ± 19.29 |
| Yes | 59.58 ± 18.71 | 58.42 ± 21.61 | 52.50 ± 21.53 | 58.33 ± 19.78 |
| p-value | 0.112 | 0.457 | **0.011** | 0.120 |
| Renal/cardiac/hepatic disorders | |  |  |  |
| No | 69.94 ± 22.91 | 67.64 ± 18.67 | 64.93 ± 26.67 | 67.81 ± 15.68 |
| Yes | 60.38 ± 18.32 | 55.56 ± 18.63 | 65.28 ± 18.63 | 59.42 ± 24.60 |
| p-value | 0.226 | 0.116 | 0.858 | 0.592 |
| Previous enteral nutrition | |  |  |  |
| No | 76.19 ± 23.77 | 66.67 ± 14.43 | 50.00 ± 33.33 | 78.82 ± 4.21 |
| Yes | 64.72 ± 21.11 | 62.72 ± 20.86 | 63.06 ± 19.97 | 62.53 ± 20.14 |
| p-value | 0.373 | 0.953 | 0.474 | 0.153 |
| Current gastrostomy | |  |  |  |
| No | 55.49 ± 22.63 | 56.49 ± 20.30 | 47.22 ± 21.65 | 56.88 ± 22.26 |
| Yes | 79.01 ± 13.42 | 69.17 ± 18.81 | 74.48 ± 12.68 | 75.00 ± 12.05 |
| p-value | **0.030** | 0.147 | **0.010** | 0.161 |

Higher the scores, higher the QoL level

Bold values: p-value < 0.05
