# Supplementary material for: Assessment of quality of life of the children and parents affected by inborn errors of metabolism with restricted diet: preliminary results of a cross-sectional study
Source: Health Qual Life Outcomes. 2013 Sep 19;11:158. doi: 10.1186/1477-7525-11-158 (PMC3848736; doi:10.1186/1477-7525-11-158)
Supplement: Additional file 3 — Children QoL: relations between scores reported by children and parents (n = 10). [file 1477-7525-11-158-S3.docx]

**Additional file 3. Children QoL: relations between scores reported by children and parents (n=10)**

| **VSP-A QoL dimensions*** | **Children [8-18 years]**  **VSP-A and VSP-Ae** | **Parents**  **VSP-Ap** | **p-value** |
| --- | --- | --- | --- |
| Relationships with family | 68.78 ± 12.25 | 71.87 ± 9.98 | 0.308 |
| Body image | 67.83 ± 19.24 | 69.17 ± 19.27 | 0.959 |
| Vitality | 72.75 ± 19.02 | 65.63 ± 15.02 | **0.038** |
| Relationships with friends | 44.50 ± 29.38 | 35.50 ± 25.33 | 0.262 |
| General well-being | 69.73 ± 15.24 | 63.23 ± 17.89 | 0.221 |
| Leisures | 39.67 ± 22.70 | 36.67 ± 17.69 | 0.372 |
| School performance | 63.75 ± 25.99 | 55.00 ± 30.16 | 0.070 |

Bold values: p-value < 0.05

Higher the scores, higher the QoL level

* Shared dimensions between the 3 questionnaires (VSP-A, VSP-Ae, and VSP-Ap)
